# Magnetic domains oscillation in the brain with neurodegenerative disease

**DOI:** 10.1038/s41598-020-80212-5

**Published:** 2021-01-12

**Authors:** Gunther Kletetschka, Robert Bazala, Marian Takáč, Eva Svecova

**Affiliations:** 1 Geophysical Institute, University of Alaska, Fairbanks, 903 N Koyukuk Drive, Fairbanks, AK USA; 2 Institute of Hydrogeology, Engineering Geology and Applied Geophysics, Faculty of Science, Charles University, Albertov 6, 120 00 Prague 2, Czech Republic; 3 1st Faculty of Medicine, Institute of Forensic Medicine and Toxicology, Charles University and General Teaching Hospital, Studničkova 4, 128 00 Prague 2, Czech Republic

**Keywords:** Neurological disorders, Chemical biology, Developmental biology, Neuroscience, Environmental sciences, Planetary science, Solid Earth sciences, Anatomy, Diseases, Astronomy and planetary science, Nanoscience and technology, Physics

## Abstract

Geomagnetic fields interfere with the accumulation of iron in the human brain. Magnetic sensing of the human brain provides compelling evidence of new electric mechanisms in human brains and may interfere with the evolution of neurodegenerative diseases. We revealed that the human brain may have a unique susceptibility to conduct electric currents as feedback of magnetic dipole fluctuation in superparamagnetic grains. These grains accumulate and grow with brain aging. The electric feedback creates an electronic noise background that depends on geomagnetic field intensity and may compromise functional stability of the human brain, while induced currents are spontaneously generated near superparamagnetic grains. Grain growth due to an increase of iron mobility resulted in magnetic remanence enhancement during the final years of the studied brains.

While the human brain contains magnetite mineralization, it has not been established if magnetism of these grains has any potential influence on the development of neurodegenerative diseases. In this work we project a new magnetic mechanism of the brain activity. The success of treatment in the above studies may relate to the presence of magnetic nanoparticles (MNPs) in the brain. The presence of the magnetic minerals in human brain has been reported decades ago^1^. Another study alerted that some of the magnetite/maghemite particles can be of external origin, coming from the polluted environment^2^. Here we focus on single domain magnetic state effect in the human brain tissue. We present room temperature, induced and remanent magnetization measurements on samples from two brains affected by neurodegenerative diseases, Alzheimer with Parkinson (B01) and Alzheimer (B02) and compared them with the brain (B03) with not known neurodegenerative disease and with the brain of the child before it was born (17 weeks—morbus Patau). We discovered that while B01 had significantly larger remanent magnetization than B02, the induced magnetization of B01 was significantly lower than B02. Both brains had significantly larger magnetic remanence compared with normal brain and the brain of the unborn child.

## Methods

### Brains

The human postmortem brains were obtained at Institute of Forensic Medicine and Toxicology and Institute of Pathology 1st Faculty of Medicine, Charles University and General Teaching Hospital between 2019 and 2020 in Prague. They were donated for examination and scientific research without time limit, in accordance with relevant guidelines and regulations in the Czech Republic. Under the laws in the Czech Republic, informed consent is not required for the collection of biological material collected during an autopsy that is anonymized and used for scientific or educational purposes. The use of these brains for scientific research was approved by the Ethical Board of the Charles University, Faculty of Science (24.4. 2020).

Brain B01 was from 79 years old male (internal ID 595/19) with Alzheimer and Parkinson disease. Brain B02 was from 80 years old female with Alzheimer disease (internal ID 474/19). Brain B03 is from 60 to 70 years old female without neurodegenerative disease (internal ID 266/20). Brain B04 was from unborn (17 weeks of pregnancy) female fetus. The removal of the brain specimens from the skull followed standard procedures. Opening of the cranial cavity was done with a saw and then was used stainless steel autopsy knife to cut off the cranial nerves and tentorium cerebelli. Spinal cord was interrupted under the brain stem to remove the brain from skull. For removing brain of the female fetus were used stainless steel scalpel and scissors. The brains were fixed in 10% formaldehyde for more than 30 days.

Each brain was first dissected into the two pieces, right (R) and left (L) hemisphere. Then each hemisphere was sliced into horizontal, 4–6 cuts. The top cut was marked as F0, and the following cuts, from the top down, as F1, F2,….Each slice was divided into two halves, front (F) and back (B). F and B slices were cut into 4–8 cm^3^ cubes, (Figure S1). Whenever possible, the orientation of the sample in respect to the scull, was preserved in the sample’s 10 ml sterile weighted plastic containers with sealable lids. Top of the container was towards the top of the scull, hinge of the container’s lid was toward the back of the scull. All cutting was done by using a ceramic knife. In this way, each sample’s location was associated with a unique identifier in three dimensions, based on its left or right hemisphere, top to down, front to back and left to right directions. Each sample was weighted. This procedure resulted in 200–300 sub-samples for each of the three adult brains, B01, B02, B03, and only two sub-samples, left and right hemisphere, from the unborn infant’s brain, B04). A few specimens were too small after cutting and had to be discarded.

### Measurement of magnetic remanence

We used superconducting magnetometer made by 2G company with horizontal sample translation, located in Pruhonice laboratory, Geological Institute, Czech Academy of Sciences. Brains samples were placed into the plastic cups and measured. Once measured we removed the sample on the porcelain plate from the plastic cup and measured the empty holder with empty plastic cup. The values of the plastic cups with holder were subtracted from the magnetic measurements. The remanent magnetization of each specimen was first measured in its initial (natural) state (called NRM for natural remanent magnetization). After the NRM value was established, the specimen was placed inside the pulse magnetizer (ASC Scientific, Model IM-10-30) with the coil exposing the sample with the magnetic pulse of 1.3 T for a few seconds. Specimen was returned to the plastic holder and its magnetic remanence was measured again in their magnetically saturated state (called SIRM, for saturation isothermal remanent magnetization); 1–2 min elapsed between SIRM acquisition and remanence measurement. Few samples had their NRM and SIRM demagnetized in steps with alternating demagnetizing field up to 20 mT (this procedure is standard part of the 2G superconducting instrument).

### Measurement of magnetic susceptibility

All collected brain samples were measured for magnetic susceptibility using magnetic susceptibility meter SM30 (Z. Hulka Inc.). It applies the AC frequency of 8000 Hz and generates field of amplitude of 40 A/m.

### Measurement of frequency dependent magnetic susceptibility

Ten samples were freeze dried inside the vacuum chamber. Freeze drying was performed in order to reduce the volume of the brain tissue so we can get measurement of frequency dependent magnetic susceptibility. Samples were measured at frequencies 4000 Hz and 8000 Hz and field of 320 A/m inside 30 ml plastic holder by instrument of SM100 (Z. Hulka Inc.).

### Measurements of X-ray fluorescence

While X-ray fluorescent analysis may be far from quantitative, we used X-ray Fluorescence (XRF) analyses (Vanta VMR by Olympus) and measured from each of the adult brain 12 randomly picked samples for measurement of major element concentration using a Geochem mode. For the unborn brain B04 we obtained for each hemisphere two chemical analyses. The instrument was used to detect the following elements along with the detection limits (ppm) in parentheses: Mg (~ 900), Al (~ 100), Si (~ 30), P (~ 20), S ~ 15), K (~ 13), Ca (~ 8), Ti (~ 4000), V (~ 12), Cr (~ 7), Mn (~ 4),Fe (~ 4), Co (~ 3), Ni (~ 2), Cu (~ 7), Zn (~ 2), Ga (~ 200), As (~ 2), Se (~ 3), Rb (~ 0.5), Sr (~ 0.5), Y (~ 2), Zr (~ 0.5), Nb (~ 2), Mo (~ 2), Ag (~ 21), Cd (~ 5), Sn (~ 5), Sb (~ 7), I (~ 40), Ba (~ 280), La (~ 450), Ce (~ 450), Pr (~ 650), Nd (~ 850), Ta (~ 250), W (~ 5), Pt (~ 65), Au (~ 2), Hg (~ 3), Pb (~ 2), Bi (~ 7), Th (~ 2), U (~ 2), and LE (~ 900). LE is the sum of the elements that are lighter than Mg. For this research we discuss only relative amounts of Fe.

Significance: Electromagnetic control of the electric noise in the human brain. Localized magnetic heating by resonance. Remote control of human brain activity. Prospects of neurodegenerative disease treatment.

## Introduction

Despite a tremendous research effort in universities and pharmacy, effective treatment of neurodegenerative diseases (ND) is lacking^3^. However, a significant advancement in magnetic resonance imaging (MRI) was established due to realization that iron concentration can be imaged by utilizing two different MRI fields on the same human subject and this gave a way to an establishment of a quantitative susceptibility mapping (QSM)^4–9^. In other words, this advancement means a realization that human brain contains nanosized magnetic compounds, including magnetic oxides with magnetization susceptible to the applied magnetic fields^1^. Dimensions of these grains are on the orders of nanometers and at the human body temperature are in so called superparamagnetic single domain state that is characterized with an enhanced magnetic susceptibility to the external field that, for magnetic iron oxides, ranges between few nanotesla to 10 s of militesla^10^. Above the militesla magnetic field range the magnetic susceptibility of magnetic oxides decreases by orders of magnitude^10^, however magnetic compounds like ferritin have paramagnetic susceptibility exceeding several Tesla range and this allowed detection of paramagnetic concentration changes when applied magnetic fields > 1 T commonly used in MRI instruments. Therefore, QSM allows detection of primarily paramagnetic (e.g. ferritin-like compounds) concentrations when applying magnetic fields exceeding 0.1 T. This is because magnetic oxides (magnetite, maghemite) are all saturated at fields > 0.1 T and contribute very little to the overall difference in magnetization^11^.

Additionally to QSM, the experiments with application of external alternating magnetic fields on human brains showed better performance on psychiatric tests^12^. Specifically, transcranial magnetic stimulation (TMS) on motor cortex^13^ delivers an alternating electric current through a coiled wire loop above the scalp. The coil, due to Ampere’s law, creates a magnetic field changes across the skull, and induces an electric currents in the brain regions due to Faraday’s law^14^.

Alzheimer disease (AD) is characterized by both deposition of senile plaques made of ß-amyloid proteins (A beta) and by hyperphosphorylation of tau proteins^15^. These formations are associated with increased iron deposits, together with changes in the regulation of iron storage in the association in neurodegenerative diseases, including AD^16,17^. So far, the antibodies aiming to modify these compounds have failed to improve cognition in clinical trials^3^. Iron is essential for normal neural function. This is because iron activates biological processes responsible for specific architecture and maintenance of neural network. Iron participates during DNA synthesis and enzymatic processes. Iron is required for fundamental brain processes that include myelination and neurotransmission. Specifically, iron is a cofactor of the iron-containing tyrosine hydroxylase that catalyzes the hydroxylation of tyrosine to form dihydroxyphenylalanine (DOPA), the precursor of dopamine, adrenaline, and noradrenaline (catecholamine neurotransmitters). Brain iron levels generally increase with the aging brain^4,18^ and show a dramatic localized increase in their brain iron content in patients with AD or Parkinson’s disease (PD)^17^. While the reason for iron accumulation in the brain in these disorders is unknown, it correlates with the production of reactive oxygen species (ROS) and oxidative damage that hallmark these disorders^17,19^.

Ionic imbalance between the ferrous (Fe^2+^) and ferric (Fe^3+^) iron seems to disrupt iron-related functions via the ROS production that often relates to the Fenton and/or Fenton-like reaction^20–22^. Divalent Fe^2+^ promotes the catalytic decomposition of hydrogen peroxide to highly cytotoxic hydroxyl radical (·OH) and trivalent form of iron Fe^3+^. Then, Fe^2+^ plays a role in the transformation reaction of H_2_O_2_ and superoxide to ·OH^23^. The ratio of Fe^2+^:Fe^3+^ in the brain of Parkinson's patients appears to be 1:3 in comparison to 1:1 in control brains^24,25^. Furthermore, in severely damaged brains of PD patients, substantia nigra contained significantly increased total Fe and Fe^3+^ ^26,27^. Oxidative stress acts via the biomolecule oxidation in the areas of the brain expressing neurodegenerative disorders of both AD and PD^20,22,28,29^.

Increased localized concentration of iron goes well with post-mortem analyses of amyloid plaques^15,30^ that revealed accumulation of copper, iron, and zinc by 5.7, 2.8, and 3.1 times, respectively, the levels observed in normal brains^3^. Additionally, it was shown that human brain tissue contains various amounts of magnetic nanoparticles (MNPs) residing in a superparamagnetic state^1,2^.

## Results

**Figure 1:**
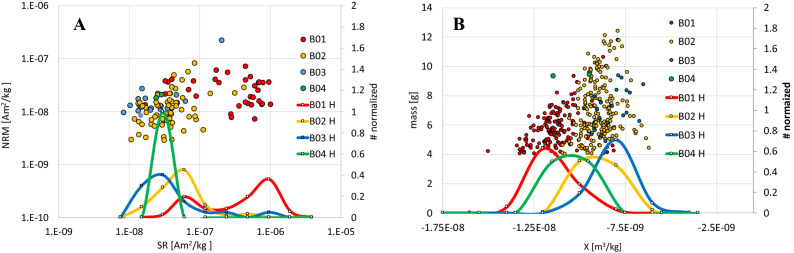
Magnetic characterization of oriented brain subsamples along with histograms. (**A**) Natural remanent magnetization and saturation remanence for the ND brains (B01, B02) and brains without ND (B03, B04) along with the frequency distribution normalize to number of samples. (**B**) Mass of the samples is plotted against magnetic susceptibility X for the brains B01, B02, B03, and B04 along with the frequency distribution normalized to number of samples.

Magnetic measurements of brain tissue were testing both occurrence of a remanent magnetization (NRM and SR) and an induced magnetization (expressed by magnetic susceptibility X). We discarded any brain samples that weighted less than 4 g. Measurements revealed an existence of MNPs in the brain capable of holding stable magnetic remanence. This is illustrated in Fig. 1A, where the spread of the NRM values for all measured brains ranged from 2.88e−9 to 2.21e−7 Am^2^/kg, and the SR values from 8.36e−9 to 1.01e−6 Am^2^/kg. The level of the natural remanent magnetization (NRM) of the brain tissues in Fig. 1 spans values from just above the noise level of the instrument (2e−9 Am^2^/kg) to more that order of magnitude above this noise level. In Fig. 1A, samples from the individual brains B01, B02, B03, and B04 were plotted in respect to level of their saturation remanence (SR). The distribution of the NRM shows that while NRM values of all brains ranged just under two orders of magnitude (B01 from 7.15e−9 to 7.03e−8 Am^2^/kg, B02 from 2.88e−9 to 8.15e−8 Am^2^/kg, B03 from 6.75e−9 to 2.21e−7 Am^2^/kg, and B04 (only two samples, see Table S1) from 1.81e−8 to 2.06e−8 Am^2^/kg), SR values distribute slightly over two orders of magnitude (B01 from 1.70–8 to 1.01e−6 Am^2^/kg, B02 from 1.08e−8 to 4.24e−7 Am^2^/kg, B03 from 8.36e−9 to 7.56e−7 Am^2^/kg, and B04 (only two samples) from 2.39e−8 to 2.87e−8 Am^2^/kg). Histograms of SR values (Fig. 1A) show a specific pattern for respective brains. Brains B03, and B04 (ND absent) show strongly overlapping distribution peaks in lower range parts of 1e−8 and 1e−7 Am^2^/kg of SR levels. The brain B02 with Alzheimer is slightly shifted from the B03 and B04 to the higher parts of SR range, just under 1e−7 Am^2^/kg SR range. The brain B01with both Alzheimer and Parkinson had significant distribution peak about order of magnitude higher than B02 near 1e−6 Am^2^/kg (Fig. 1A).

Figure 1B shows how mass of the brain samples varied with the induced magnetization (magnetic susceptibility X). Brain B01 had slightly narrower mass (volumes) distribution than samples of brains B02, B03 and B04. The levels of X showed range between − 1.50e−8 and − 6.18e−9 m^3^/kg. The susceptibility frequency distribution had the maximum near − 1.3e−8 m^3^/kg for B01, followed by maximum near − 1.05e−8 m^3^/kg for B04, then maximum near − 9.5e−8 m^3^/kg for B02 and then maximum near − 8.0e−9 for B03. Specifically, the magnetic susceptibility ranged between − 1.50e−8 and − 8.12e−9 m^3^/kg for B01, between − 1.13e−8 and − 6.18e−9 for B02, between − 9.33e−9 and − 6.48e−9 for B03 and between − 1.23e−8 and − 9.02e−9 for B04.

Since magnetic susceptibility is often driven by concentration of iron, we compared X values and its error distribution with the XRF detected concentration of iron (Figure S2B) and showed that the iron concentration varied between 20 and 230 ppm, specifically B01 20–80 ppm, B02 25–225 ppm, B03 55–125 ppm, and B04 5–35 ppm (Figure S2 and XRF DATA SI.xlsx file in supplementary material).

Demagnetization of brain samples’ NRM with alternating field up to 40 mT showed no significant decrease in magnetic remanence (Fig. S3). This suggests the values were close to the detection limit of the superconducting magnetometer and such data need to be taken with caution. While working near the detection limit, the direction of the magnetic remanence had no random distribution (Fig. S4). The overall direction of the brain magnetization was from the front to back. The precision of the direction preservation when inserting the brain fragments into the plastic holders was estimated as better than 40-degree cone and this may have contributed to the large spread of direction distribution.

**Figure 2:**
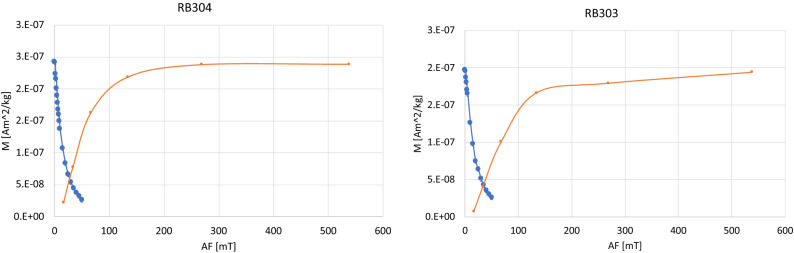
Typical acquisition of magnetic remanence and demagnetization of saturation remanent magnetization of brain B01 samples. Error bars are 2e−9 Am^2^/kg.

Magnetic nanoparticles in the brain can have either homogeneous distribution, or they may be in clusters of particles. Detection of magnetic nanoparticles interaction relates to how easily samples are magnetized and demagnetized. This analysis is shown in Fig. 2 for brain B01. During the magnetizing samples towards the saturation, half of the saturation level was typically reached using exposure of the sample to ~ 50 mT magnetic field pulse from pulse magnetizer. Brain samples become magnetically saturated when exposed to the 100–200 mT magnetic pulse. After magnetic saturation brain samples showed steep magnetization decay, and when exposing them to alternating field between 10 and 15 mT they demagnetized to 50% of the saturation level (Fig. 2). The amount of saturation remanence imposed by pulse magnetic field was 1075 mT (ASC—pulse magnetometer).

The brain tissue contains superparamagnetic grains^1,2^. This is evidenced not only by 5–6-min rapid magnetization decay by 0.5% (Figure S5), but also by decrease in magnetic susceptibility when increasing frequency from 4 to 8 kHz (Figure S6).

## Discussion

SR data (Fig. 1A) showed remarkable distinction between ND brains (B01, and B02) and the brains without ND (B03, and B04). Brains without ND show low level of SR and indicate that magnetic nanoparticles, if present, are not capable of holding magnetic remanence. Brains with ND, both showed significant increase in ability to hold SR. While the B02 with AD had increased SR values to its maximum just under 1e−07 Am^2^/kg, similar maximum has been detected for B01 brain with not only AD but also PD. Brain B01 with PD, in addition to the peak coexisting with the SR peak from B02, shows more significant peak just under 1e−6 Am^2^/kg. Magnetic carriers in B01 are likely from the transition between single domain magnetic state and superparamagnetic state. Superparamagnetism is indicative by evidence of frequency dependent susceptibility measurement of B01 sample that shows lower magnetic susceptibility for frequency measured at higher frequency (Figure S6). In addition, Fig. 2 suggests interacting magnetic particles (non-interacting grains would cross at 50% of the magnetization level) and this indicates that the distribution of these grains is in clusters near each other. The increasing/decreasing external magnetic field is superimposed significantly with the magnetic fields from the neighboring magnetic grains^31^. The SR levels of B01 and B02 were higher (Fig. 1A) and are indicative of larger magnetic particles in these brains, compared with brains B03 and B04.

**Figure 3:**
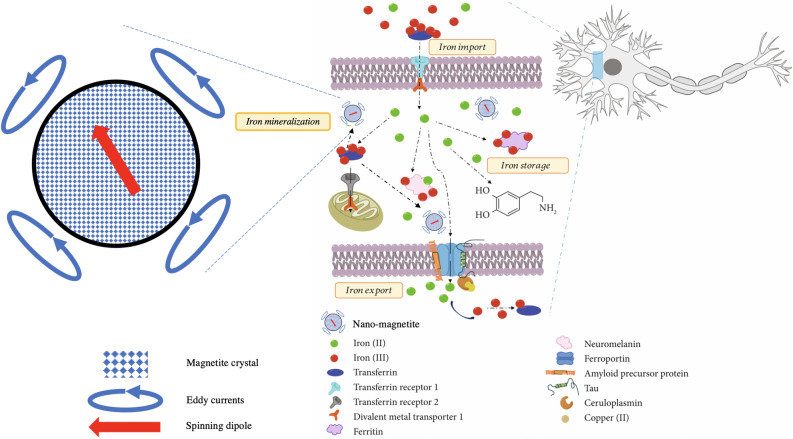
Iron metabolism in the neuron (modified from Abeyawardhane and Lucas (2019)^21^).

Indication of iron mineralization in the brain along with observation of magnetic interaction adds more details to the indication of inhibition of iron-export ferroxidase activity of ß-amyloid precursor protein^15,32^ that may lead to AD^30^. While evidence of disruption of such pathways was indicated to lead to overcrowding of iron molecules^32^ it was not clear where and how such overcrowding process materializes. The specific details how the iron ions participate in iron metabolism and how overcrowding of these ions may lead to magnetic iron mineralization are outlined in Fig. 3. Dysfunction and/or equilibrium change between ferric and ferrous ions can initiate due to Fenton chemistry associated ROS generation^21^. Iron accumulation is due to transferrin (Tf), main iron transport glycoprotein in the central nervous system (CNS)^33^. Tf has ferric iron binding sites where these ions get engulfed into cells via Tf receptor-1 (TfR-1) using endocytosis^34^. Ferric ions inside the neurons get to be reduced to ferrous and get into the cytosol via divalent metal transporter-1 (DMT-1). The concentration of iron ions in neurons is kept in balance by iron regulatory proteins (IRP) as a feedback from activity of TfR-1 and DMT-1 (Fig. 3). When the neural cell is iron deficient, IRP allows iron increase by activating the mRNA coding for both TfR-1 and DMT-1^35^. This is done by binding to iron responsive element (IRE) that forms a 26–30 nucleotides loop structure that commonly occurs in 3′ or 5′ untranslated regions (3′-UTR or 5′-UTR) of eukaryotic mRNA that is responsible for balancing translational iron dependency^21^. There is another iron transporter in neural cells, transferrin receptor-2 (TfR-2) that has missing IRE and is more prevalent in the mitochondria of neural cells^36^. While mitochondrial dysfunction was observed hand in hand with elevated Tf and TfR-2 levels, it suggests an oxidative stress promoted by iron redox chemistry^34,36^. Intracellular iron is also balanced by translation of ferritin, the most significant iron storage protein in the 5′-UTR of Tf mRNA^37^, and neuromelanin^38^. Both ferritin and neuromelanin has been observed to contain dense iron cores^39^, which may allow for mineralization of magnetic iron oxides observed here (Fig. 3).

Magnetic levels of SR (Fig. 1) support that iron mineralization is taking place in the brain with ND and that the mineralization leads to clusters of magnetic minerals, unevenly distributed in the brain with ND. Note that while B01 showed high SR levels at the same time this brain has lowest levels of magnetic susceptibility (Fig. 1B) that is a common measure for iron concentration constrained also by XRF iron concentration analysis. Thus, we have evidence that while the published work^17^ and B02 have concentration of iron higher we also have evidence of iron concentration in B01 is being lower than normal B03 (Figure S2B). It appears that Zinc plays a critical role in the iron export inhibition^32^ as partly supported by XRF composition analyses of brain samples (Figure S2C). Figure S2C supports that while B01 shows the maximum SR levels it also shows maximum level of zinc. Note, however, that the same B01 indicates the lowest amount of iron. This observation suggests that the iron pathway is disrupted in a way that iron becomes in some parts of the brain diluted and thus insufficient to take part in the RNA for the iron importer transferrin receptor (TFR) mRNA in the 5′ untranslated region (UTR) of APP that mRNA possesses^40,41^. While iron dilutes, it raises the concentration level in the brain’s blood flow and this imbalance may have a feedback in iron mineralization in the brain leading to the increase of magnetic remanence properties (Fig. 1).

Note that in AD, Zn2+ accumulates with ß-amyloid (Ab) in extracellular plaques, hallmark for AD^40^. A broadly expressed type I transmembrane protein precursor (APP) of uncertain function generates Ab^41^. A functional IRE stem loop has sequence homology to the IREs for ferritin and TFR mRNA in the 5′ UTR of APP^40,41^. Such APP translation would be sensitive to free iron levels in cytoplasma. In addition, APP translation also controls the way how iron regulatory proteins bind to TFR mRNA and ferritin itself^42^. With high cellular iron levels, translation of AD ß-amyloid protein precursor and the iron-storage protein ferritin would increase^41^, while RNA for the iron importer TFR would degrade. Our data in Fig. 1 and Figure S2 suggest that low level of iron is associated with high level of zinc for B01, while there is a clear evidence of increased iron mineralization via detecting the ability of the brain samples to hold a saturation remanence (Fig. 1A). While such an association connects AD with zinc, it also suggests that both low and high iron levels can occur in ND brain (we see the low level of magnetic susceptibility and low concentration iron levels by XRF obtained for B01 in Fig. 1B, Figure S2A, S2B). XRF also revealed somewhat low levels of Ca (Figure S2D) that may be a sign of dysregulation of Ca2+ buffer expression that is associated with neurologic/neurodevelopmental disorders^43,44^.

Data in Figure S4 revealed non-random distribution of natural magnetic remanence in the studied brain (B01). However, note that the NRM levels were all near the limit of the instrument resolution. Interestingly, the magnetic direction was from a front to a back of the brain. The person’s medical history indicates that the person spent the last few years lying in horizontal position in bed. Such position exposes the brain to the natural geomagnetic field direction which is close to vertical, between 60° and 70° from horizontal, in the Prague’s magnetic altitude. Observation of front to back magnetic remanence suggests that most of the growth of magnetic particles was occurring at the time when brain carrier was in the horizontal position with brain directed more or less parallel to the geomagnetic field. Such magnetization acquired by grain growth is known as chemical remanence^11^.

### Theory of magnetic remanence acquisition

Chemical magnetization (CRM) is recorded at the blocking volume V_B_ when a magnetic mineral grows in an applied field H_0_. The ratio of CRM *M*_*cr*_ of a single domain (SD) grain of volume *V* that is characterized by a saturation magnetization *M*_*s*_ at blocking temperature *T*_*B*_ and coercive force *H*_*C*_ is the same as theory for thermoremanent magnetization (*Néel* 1949) only by using *M*_*cr*_ instead of thermoremanent magnetization (M_tr_).

1 $$\frac{{M}_{cr}}{{M}_{rs}}=tanh\frac{{\mu }_{0}V{M}_{s}\left({V}_{B}\right){H}_{0}}{kT},$$

where ${M}_{rs}$ is the saturation remanent magnetization, ${\mu }_{0}$ is the magnetic constant, and *k* is the Boltzmann’s constant. From the Néel’s (1949) theory, we have the relationship that describes the timescale τ by which magnetic remanence is acquired at the blocking temperature for specific volume *V* (analogy to blocking volume V_B_ for specific temperature T in our case).

2 $$\frac{{\mu }_{0}V{M}_{s}\left({V}_{B}\right){H}_{c}({V}_{B})}{2kT}=\mathrm{ln}\left(\uptau /{\uptau }_{0}\right),$$

where τ_0_ is a characteristic timescale of thermal oscillations, which is approximately 10^−9^ s. For laboratory experiments where we record magnetic remanence τ is generally on the order of 100 s or more, which gives ln(τ/τ_0_)≃25. Due to the logarithmic dependence on time, ln(τ/τ_0_) is relatively insensitive to the time scale of magnetization acquisition. Solving for M_s_ in the above equation and inserting into Eq. (1) provides the relation.

3 $$\frac{{M}_{cr}}{{M}_{rs}}=tanh\frac{2\mathrm{ln}\left(\uptau /{\uptau }_{0}\right) {H}_{0}}{{H}_{c}({V}_{B})}.$$

For superparamagnetic cases the natural remanent magnetization M_cr_ of individual grains is equal that of M_rs_, and we can simplify this equation to

4 $$1=tanh\frac{2\mathrm{ln}\left(\frac{\uptau }{{\uptau }_{0}}\right) {H}_{0}}{{H}_{c}\left({V}_{B}\right)}$$

This approximately satisfies (4) for all arguments of tanh function that are greater than 2. Using the boundary value of 2 we have:

5 $$2{H}_{c}\left({V}_{B}\right)\sim 2\mathrm{ln}\left(\frac{\uptau }{{\uptau }_{0}}\right) {H}_{0}$$

6 $$\frac{{H}_{c}\left({V}_{B}\right)}{{H}_{0}}\sim \mathrm{ln}\left(\frac{\uptau }{{\uptau }_{0}}\right)$$

Then for the time scales of electric currents $\uptau \sim {10}^{-1}s$ in human brain and thermal fluctuations ${\uptau }_{0}\sim {10}^{-9}s$ we have

7 $$8{H}_{0}\sim {H}_{c}\left({V}_{B}\right).$$

Result in (7) suggests that, for the human brain’s frequencies of electrical current, the coercivity of magnetic grains at the temperature (36 C) is the field in which the magnetic grains grew (e.g. geomagnetic field ~ 0.05 mT) multiplied by eight. This calculation constrains an estimate of the magnetic field magnitude required to destabilize the characteristic magnetic remanence blocking fluctuating at frequencies of 10 Hz. If the fluctuation of magnetic moments interferes with the brain function, application of fields of this magnitude and frequency would directly control the interaction of the MNPs that has the specific size (generates the 10 Hz frequency) with the brain’s synapses. This opens a new way how the parts of the brain with iron mineralization to the specific size (having specific H_c_(V_B_) in (7)) of magnetic carriers could be controlled by application of electromagnetic pulses of specific amplitude and frequency.

Magnetization of magnetite particles creates space around them where magnetic field decays exponentially from 200 to 2 mT^45^. Taking 20 mT magnetic field from this range changing its direction with the frequency determined by the size of the magnetic grain allows estimation of the electric currents generated within the brain tissue by these magnetic grains. Because the frequency of the synapses’ electrical signatures is between 0.1 and 500 Hz^46^ we take frequency of 10 Hz from this range of frequencies, like we did in (8). Human brain resistance in high frequencies reaches 10 Ohms^47^. The currents that is capable of repeatable neural synapses’ activation is near 100 pA^48^. Magnetic field in vicinity of single domain magnetic grain reaches 100 s mT in a spherical volume of 60 nm in diameter (superparamagnetic sphere of 20 nm in diameter). Presence of magnetic particles in the brain represents a hotspot for generation of an alternating currents near the MNPs that may be near synapses. For Faraday’s law we have

8 $$I=\frac{A}{R}Bf,$$

where R is the brain’s resistance, A is the area of fluctuating magnetic field B, and f is the frequency. While the current estimate from such system is in fA range, it may catalyze the charge distribution along the neural synapses.

Note that because the MNPs are within a conducting medium, the generation of current would resist the magnetic moment fluctuation. In reality this would decrease the frequencies of small superparamagnetic grains and create potential for magnetic tunneling^49^.

### Iron mineralization

Iron regulatory proteins, ferritin, and neuromelanin, all serve for an iron storage. Evidence of biomineralization of MNPs in the brain is likely due to disruption of the iron pathways, perhaps due to free zinc presence^32^. The exact MNPs location in terms of the cellular neural function is not known. When MNPs are small, induced currents from fluctuating magnetic dipoles distribute in only small volumes and the frequency of these currents (> 500 Hz) is outside the frequency of neural tissue^46,50^. However, as the MNPs grow, their increased fluctuating magnetic moments and decreased frequency, generate microcurrents that start matching the frequency of neural connection between 1 and 500 Hz^46,50^. Such interference may result in neural connection malfunction and may contribute to the disruption of the iron pathways^32^. This is because the accumulation of the MNPs often collocates with places of the neurotransmitters^17^.

Hysteresis loop on brain samples showed that ND contains single domain grains apart from superparamagnetic grains^51,52^. Magnetic properties detected by SQUID magnetometer attributed the magnetic signature to the presence of magnetic iron oxides^52^. Hysteresis properties allow an estimation of a concentration of single domain magnetic grains per mass. Figure 1 shows that the saturation remanence of the measured B01 sample ranged between 1.8 to 3.7 e−7 Am^2^/kg. Given that single domain size for magnetite/maghemite is 100 nm^11,53^ and that magnetite/maghemite have saturation magnetization constants 93.2 and 77.6 Am^2^/kg, respectively we use average value 85 Am^2^/kg. Saturation remanence of SD grains is about 1/2 of saturation magnetization, leaving 47.5 Am^2^/kg compared with measured 1.8 to 3.7 e−7 Am^2^/kg ~ 2.7 e−7 Am^2^/kg gives concentration 5.8 ng/g. Comparison with concentration of 1000 s of nanograms per gram reported in AD tissues from hysteresis measurements^54^ indicates that only 0.5% of the magnetic iron was capable of carrying magnetic remanence in B01. This leaves the rest of the iron being in superparamagnetic state with their magnetic vectors fluctuating with high frequencies that decrease with increasing size^49^.

The electric activity of the brain ranges between 0.1 and 500 Hz^46^. There are variations in electric activity spectra where some peak location (e.g. around 10 Hz) seems to be relevant for dementia and AD^50^.

### Possible relation to ND

Our data for brain tissue without ND agree with published reports indicated that healthy subjects without ND have magnetic grains mostly in superparamagnetic state^1,2^, for which the grain sizes are such that they do not interfere with the brain frequencies of < 500 Hz, and their concentration increase result in increase of magnetic susceptibility. Such magnetic grains have sizes of less than 40 nm^11^. Magnetic moments fluctuate in broad spectrum of frequencies, ranging from over nine orders of magnitude, from high (10^9^ Hz) down to units of Hz. Our measurements showed that the brain with ND is associated with not only an increased iron concentration, but also biomineralization of nano-sized magnetic iron oxides and growth into their single domain magnetic state. These iron oxides likely nucleated from the solid iron cores that have been observed inside ferritin and neuromelanin^39^. Magnetic iron oxides serve as a new source of high frequency electric currents inside the neuron structures. Our analyses showed that the ND brains have increased volume of these magnetic oxides that not only slows down the frequency down to frequencies of neural transmissions (10 Hz) but also increase the volume of the neural tissue that would be affected by magnetic nano-oxides’ eddy currents. Evidence of this new electric source stems from detection of magnetic grain remanence indicating the presence of magnetic grains that already exceeded 100 nm grain size. Once the grains grow into the single domain magnetic state, their mass normalized magnetic susceptibility decreases and this is what we have detected (Fig. 1).

This newly identified potential mechanism of iron interference with the neural functions does not uniquely single out that iron accumulation is the cause of ND but it strongly supports it. Such novel mechanism suggests that iron accumulation in human brain is the cause of magnetically stimulated neurodegenerative disease rather than a consequence of it, answering the question that was focus of multiple studies that used quantitative iron levels^55^ and quantitative iron magnetic susceptibility mapping (QSM)^8,9,56^. Magnetic susceptibility has become relevant to the study of aging^4,57^ and ND due to high contrast in QSM using magnetic resonance imaging (MRI)^6,55,57–59^. While QSM focused on monitoring the spatial distribution and the temporal dynamics of iron deposition to gain insight of our understanding of ND pathogenesis^7,15^, it could not resolve if the iron accumulation is the cause or the effect^6–9,15,19,55–57,60,61^, despite, for example, superior AD identification compared to MRI analyses with gray matter volume changes^61^.

If iron homeostasis is significantly disrupted, it may lead either to iron accumulation^22^ or potential iron disappearance^15^. Such iron concentration changes, in terms of their grain sizes, could constrain novel reasoning, why there is a general disagreement between the brain iron concentration and gray vs white matter, that is currently attributed to an increase in myelin content rather than concentration of iron^5,6^. While iron accumulation might constitute ideal proliferation and perpetuation environments for ß-amyloid aggregation and neurotoxicity^15,30,62,63^, our data support that both iron accumulation and reduction may lead to a significant increase in magnetic particle size, generating the electric currents that may interfere with the normal neural functions.

## Conclusions

We show that a nano-mineralization of magnetic grains in human brains corelates with the progression of neurodegenerative diseases. Magnetic nanoparticles contain oscillating magnetization that in conducting brain tissue generates electric currents that may interfere with the synapses function in the brain. The progress of mineralization appears to be significantly enhanced in brains with neurodegenerative disease (ND). This observation proposes a new phenomenon that may interfere with the normal brain processes. Due to nanosized volume, magnetic moment oscillates within the MNPs with frequency up to 1e9 Hz. Microcurrents generated this way interfere with the brain cellular functions. As the iron in the brain migrates, perhaps due to Zinc interrupting the iron pathways, the MNPs volume increases from the nuclei made from ferritin and neuromelanin precursors. This increase in iron oxides’ volume broadens the frequency of induced electric currents (IECs) from gigahertz down to sub-hertz frequencies. When frequency of IECs matches the frequency of electric currents of synapses, the two currents may interfere via resonance and this may contribute to neural malfunction that contributes to the form of neurodegenerative disease (ND).

Our data revealed a new phenomenon that explains why there can be both lower and higher magnetic susceptibility in brains with ND. While most of the magnetic grains that grow in the brain would be superparamagnetic in early onset of ND and the susceptibility increases with this progression, when the grains grow into the single domain magnetic states the susceptibility gets lower.

We postulated a new mechanism for electric interference of MNPs with the neural synapsis function. Theoretical consideration of blocking the remanence direction fluctuating in the brain during the MNPs growth identified that magnetic coercivity of MNPs are sensitive to geomagnetic field magnitude.

## Supplementary Information

Supplementary Information
