# Supplementary material for: Magnetic domains oscillation in the brain with neurodegenerative disease
Source: Sci Rep. 2021 Jan 12;11:714. doi: 10.1038/s41598-020-80212-5 (PMC7804002; doi:10.1038/s41598-020-80212-5)
Supplement: Supplementary file 1 — Supplementary Information [file 41598_2020_80212_MOESM1_ESM.docx]

**Magnetic domains oscillation in the brain with neurodegenerative disease**

Supplementary Material

Gunther Kletetschka^1,2,3^, Robert Bazala^2^, Marian Takac^2^, and Eva Svecova^2^

1. Geological Institute, Czech Academy of Sciences, 165 00 Prague 6, Rozvojová 269; Czech Republic;

2. Department of Applied Geophysics, Charles University, Albertov 6, 120 00 Prague 2, Czech Republic;

3. Geophysical Institute, University of Alaska, Fairbanks, 903 N Koyukuk Drive, Fairbanks, AK, USA,

4. Institute of Forensic Medicine and Toxicology, 1^st^ Faculty of Medicine, Charles University and General Teaching Hospital, Studničkova 4, 128 00 Prague 2, Czech Republic

Corresponding author: Gunther Kletetschka, [kletetsg@natur.cuni.cz](mailto:kletetsg@natur.cuni.cz)

Figure S1: Map of B01 subsamples. Numbers (00, 01, …, 08) assigned to each of the maps represent horizontal slices of the brain. Map divides artificially brain into front of the brain (upper quadrangles) and back of the brain (lower quadrangles). Inset photographs shows the relation between the map and brain subsamples.

A. B.

C. D.

Figure S2: Induced magnetization by measurement of magnetic susceptibility and XRF composition.

Figure S3: Typical demagnetization of natural remanent magnetization of brain samples. Error bars are 2e-9 Am^2/kg


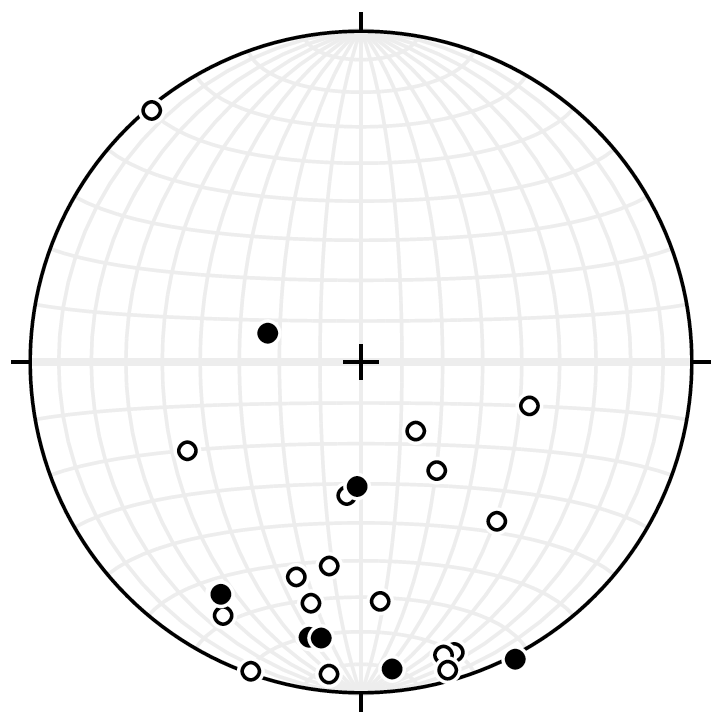


Figure S4: Directions of magnetic remanence from oriented samples from B01 (see Figure S1 for exact locations: LB511, LB514, LB522, LF511, LF512, LB602, LB603, LB604, LB611, LB613, LB703, LF602, LF612, LB704, LB713, LB714, RB303, RB304, RB312, RB321, RB322, RB323, RB331, and RB302). Up is towards the nose direction. Down is towards the back of the head. Middle cross is vertical. When dots are solid, the direction is towards legs, when empty, direction is towards above the head.

Figure S5: Magnetic decay of brain sample while in magnetic vacuum <200 nT.

Figure S6: Measurement of susceptibility at two different frequencies, 4 kHz and 8 kHz. Samples in Supplement Table 1 were packed together into one. Each frequency was measured 1000 times.

Table S1: Statistical parameters (N- number of samples, a-average, std-standard deviations, min-minimum value, max-maximum value) of the natural remanent magnetization (first column) and saturation magnetic remanence (second column) measurements for B01, B02, B03, and B04, respectively.

Table S2: Statistical parameters (N- number of samples, a-average, std-standard deviations, min-minimum value, max-maximum value) of the magnetic susceptibility measurements for B01, B02, B03, and B04. For sample B04 there were 2 samples, each measured 6 times.

Table S3. Freeze dried brain samples combined for measurement of frequency dependent magnetic susceptibility. Masses of the subsamples are shown before and after freeze drying.

| Sample | Initial mass [g] | Frees dried mass [g] |
| --- | --- | --- |
| RF321 | 5.376 | 1.197 |
| RB311 | 7.323 | 2.299 |
| RB414 | 8.905 | 2.658 |
| LF513 | 5.774 | 1.805 |
| RF323 | 8.048 | 2.512 |
| RB311 | 6.211 | 2.705 |
| RB332 | 6.218 | 1.888 |
| RF322 | 6.450 | 1.531 |
| RB404 | 8.187 | 1.695 |
| RB413 | 7.722 | 1.867 |

Table S4: X-ray Fluorescence data on B01, B02, B03, and B04 (Excel file XRF DATA SI.XLSX)
